# Flow‐based immunomagnetic enrichment of circulating tumor cells from diagnostic leukapheresis product

**DOI:** 10.1002/1878-0261.13565

**Published:** 2024-01-18

**Authors:** Michiel Stevens, Anouk Mentink, Frank A. W. Coumans, Eshwari Dathathri, Khrystany T. Isebia, Jaco Kraan, Ronald de Wit, John W. M. Martens, Leon W. M. M. Terstappen

**Affiliations:** ^1^ Department of Medical Cell BioPhysics, Faculty of Science and Technology University of Twente Enschede The Netherlands; ^2^ FETCH BV Deventer The Netherlands; ^3^ Decisive Science Amsterdam The Netherlands; ^4^ Department of Medical Oncology Erasmus MC Cancer Institute Rotterdam The Netherlands; ^5^ Department of General, Visceral and Pediatric Surgery University Hospital Düsseldorf Germany

**Keywords:** circulating tumor cells, diagnostic leukapheresis, flow enrichment, magnetic enrichment

## Abstract

The clinical utility of circulating tumor cells (CTCs) is hampered by the low number of cells detected. Diagnostic leukapheresis (DLA) offers a solution but, due to the observed non‐specific binding and clumping, processing of DLA samples using the CellSearch system only allows for the processing of aliquots consisting of ~ 2% of the total DLA sample per test. Here, we introduce a flow enrichment target capture Halbach‐array (FETCH)‐based separation method in combination with a DNase preprocessing step to capture CTCs from larger fractions of DLA products without clumping. To evaluate the FETCH method, we processed peripheral blood samples from 19 metastatic castration‐naïve prostate cancer (mCNPC) patients with CellSearch, and processed 2% aliquots of leukapheresis samples from the same patients with CellSearch as well as FETCH with or without DNase preprocessing. Using 2% aliquots from six patients, the use of FETCH with fewer immunomagnetic epithelial cellular adhesion molecule (EpCAM) conjugated ferrofluids was tested, whereas 20% aliquots from four patients were used to evaluate the processing of 10‐fold larger DLA samples using FETCH. Results show that the cell clumping normally seen after immunomagnetic enrichment of DLA material was greatly reduced with the use of DNase pretreatment, while the number of CTCs detected was not affected. The number of CTCs detected in 2% aliquots of DLA using FETCH was unchanged compared to CellSearch and did not decrease when using down to 10% of the volume of immunomagnetic anti‐EpCAM ferrofluids normally used in a CellSearch test, whereas the number of co‐enriched white blood cells reduced a median 3.2‐fold. Processing of a 20% aliquot of DLA with FETCH resulted in a 14‐fold increase in CTCs compared to the processing of 2% aliquots of DLA using CellSearch and a total 42‐fold median increase in CTCs compared to peripheral‐blood CellSearch.

## Graphical abstract

The number of circulating tumor cells obtained from prostate cancer patients was increased approximately 5‐fold compared to regular CellSearch when processing 2 mL diagnostic leukapheresis material aliquots and increased by 44‐fold when processing 20 mL DLA aliquots using the flow enrichment target capture Halbach‐array. Clumping of cells during the procedure could be prevented by pre‐incubation with DNase.

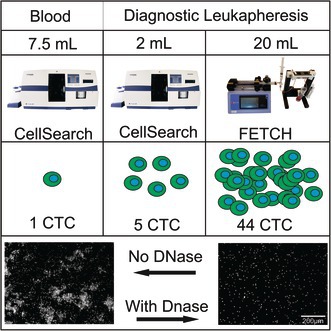

**Abbreviations**
CTCs: Circulating tumor cells
DLA: Diagnostic leukapheresis
EDTA: Ethylenediaminetetraacetic acid
EpCAM: Epithelial cell adhesion molecule
FETCH: Flow enrichment target capture Halbach‐array
mCNPC: metastatic castration‐naïve prostate cancer
mPC: metastatic prostate cancer
PBS: Phosphate‐buffered saline
WBCs: White blood cells

## 1 Introduction

The median number of Circulating Tumor Cells (CTCs) currently detected by the CellSearch system in 7.5 mL of whole blood in early‐stage cancer patients is 0 or 1 [1, 2, 3, 4]. This is too low to effectively monitor and guide treatment in all patients. A statistical modeling study suggests that a minimal 15‐fold increase in CTC recovery would be needed when aiming to detect CTCs before the occurrence of distant metastasis [2], while another modeling study shows that a more than 500‐fold increase in sampling volume is needed to detect EpCAM‐enriched CTCs in 99% of metastatic cancer patients [5]. Clearly, the number of CTCs found needs to be increased, either by capturing CTCs that are currently missed by CellSearch or by increasing the sample size. A substantial increase in sample size can be achieved by the use of DLA [6], which allows for increased detection of CTCs [7]. However, while the mononuclear cell fraction of up to 10 liters of blood can be collected, due to assay limitations in CellSearch the DLA sample volume per test is equivalent to only 115 mL of whole blood, yielding a 5‐ to 6‐fold increase in CTCs [8, 9]. This limitation is due to the excessive number and clumping of white blood cells (WBCs) that are co‐enriched in the immunomagnetic enrichment procedure, hampering the efficient identification of tumor cells in the CellSearch identification procedure [10].

The use of DNase to remove free genomic DNA is known to prevent clumping during thawing of cryopreserved WBCs [11] and assist in the generation of single‐cell suspensions [12, 13]. This has however not been used for the prevention of clumps formed during the immunomagnetic enrichment of rare cells. As free DNA might be present at higher levels in DLA products compared to blood due to a small portion of dead or apoptotic cells among the high concentration of WBCs, we expected the involvement of free genomic DNA in forming these clumps. Additionally, the number of WBCs co‐enriched is dependent on the specificity of the magnetic labeling and used method for magnetic separation. We have previously optimized a magnetic configuration to more efficiently capture cells with lower amounts of magnetic particles [14]. Here, we introduce the use of DNase for the prevention of cell‐clumping during immunomagnetic enrichment of CTCs from DLA material in combination with a Flow Enrichment Target Capture Halbach array, or “FETCH,” and compare its efficiency for the selective capture of CTCs from DLA samples obtained from mPC patients to that of CellSearch.

## 2 Materials and methods

### 2.1 Patient samples

Blood and DLA samples were obtained between February 2022 and April 2023 from 19 metastatic Castration Naïve Prostate Cancer (mCNPC) patients before initiation of treatment and with > 2 CTCs detected by standard CellSearch in a 7.5 mL sample of blood. One patient underwent a second leukapheresis procedure after becoming castration‐resistant, resulting in 20 samples. Blood draws and leukapheresis were performed at the Erasmus Medical Center in Rotterdam, the Netherlands. Leukapheresis was performed per the optimized procedure described by Mout et al. [15] on a Spectra Optia (Terumo, Lakewood, CO). Both blood and DLA samples were stored in CellSave vacutainers prior to processing. Samples were collected in accordance with the Declaration of Helsinki as part of the PICTURES study approved by the medical ethical committee of the Erasmus Medical Center (MEC20‐0422). All patients signed informed consent prior to any study procedure. An overview of the cell composition per mL, including the detected CTCs, of the obtained DLA products is shown in Table S1.

### 2.2 Flow enrichment target capture Halbach‐array (FETCH)

**Fig. 1:**
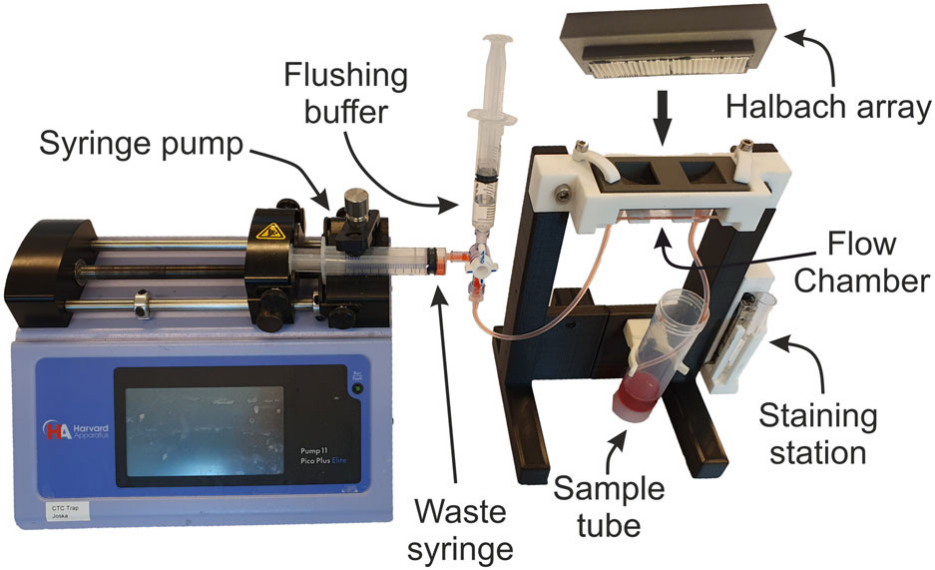
Image of FETCH device. Image of the FETCH magnetic separation device used for the capture of magnetically labeled CTCs from DLA material.

The FETCH magnetic separation device shown in Fig. 1 consists of an optimized Halbach array [14] against which an Ibidi μslide flow chamber with a height of 0.8 mm, a width of 5 mm, and a length of 50 mm is placed (Ibidi, Gräfelfing, Germany). A syringe pump equipped with a 10 mL waste collection syringe is used to pull the sample from the sample tube through the flow chamber. The waste syringe is connected to the flow chamber outlet through tubing with an inner diameter of 1.5 mm. The same 1.5 mm inner diameter tubing is connected to the flow chamber inlet and is placed into the sample tube, allowing the sample to be pulled through the chamber. After separation of the sample and flushing of the flow chamber, the tubing is diverted to the collection tube and the Halbach array is removed. The tubing leading to the waste syringe is then diverted to a 5 mL syringe containing 2 mL of buffer and the enriched sample is collected by pushing through 2 mL of buffer alternated with air to achieve complete sample removal. The collected volume of the immunomagnetically enriched cells is 2.5 mL. Although the small diameter tubing and shallow flow channel prevent high levels of sedimentation, the enriched fraction was magnetically washed after flow separation to remove any remaining unbound cells before immune‐fluorescent labeling.

### 2.3 Enrichment

Whole blood collected in CellSave preservative tubes was enriched using the CellSearch protocol. DLA samples were fixated by placement in CellSave preservative tubes and enriched using both FETCH and CellSearch.

#### 2.3.1 CellSearch

A modified CellSearch protocol was used for DLA samples. Aliquots of 2 × 10^8^ WBC (2%‐DLA) were placed in conical tubes and supplemented to 11 mL with CellSearch dilution buffer. Samples were then centrifuged at 800 ***g*** for 10 min. The CellSearch Autoprep red cell detection system was tricked using black tape, and approximately 6 mL was left behind during immunomagnetic enrichment. The other steps were the same as the standard CellSearch protocol.

#### 2.3.2 FETCH

DLA aliquots of 2 × 10^8^ WBCs (2%‐DLA) were diluted to 5 mL using PBS buffer (Merck, Darmstadt, Germany) supplemented with bovine serum albumin (Merck), EDTA (Merck), casein (Merck), and mouse serum (Invitrogen, Carlsbad, CA), from here on referred to as “CellBuffer.” DLA aliquots of 5 × 10^8^ WBCs (5%‐DLA) were depending on their initial volume either diluted using CellBuffer or concentrated using centrifugation at 400 ***g*** to 5 mL. DLA aliquots containing 2 × 10^9^ (20%‐DLA) were concentrated to 10 mL using centrifugation at 400 ***g***. Next, in the case of DNase pre‐processing, DNase I (Roche, Basel, Switzerland) was added to a final concentration of 20 μg·mL^−1^ and activated with MgSO_4_ (Sigma‐Aldrich, St. Louis, MO) at a final concentration of 20 μm, after which the sample was incubated at RT for 5 min. Then, 25‐ to 150 μL of CellSearch ferrofluid was added and incubated for 3 min, after which an equal volume of CellSearch capture enhancement was added, and the sample was incubated in a quadrupole magnet for seven times 3 min, mixing the sample by inversion between incubations.

Separation was performed by pulling the sample through the Ibidi μslide flow chamber against which an optimized Halbach array was positioned. Sample speed was controlled at 0.5 mL·min^−1^ using a syringe pump (Harvard Apparatus, Holliston, MA). After the sample passed, the flow chamber was washed using 1 mL of CellBuffer at the same flow speed to remove residual uncaptured cells. Next, the magnetic array was removed and the collected sample was retrieved by flushing the chamber with 2 mL CellBuffer and air using a manually operated syringe. Enriched samples were collected in a 12 × 75 mm conical tube and magnetically separated for 5 min in an iMAG magnet (BD, Franklin Lakes, NJ). The unbound fraction was then aspirated using a glass Pasteur pipet and the syringe pump set to 2 mL·min^−1^. Samples were washed using 1 mL PBS BSA 1%, separated again and resuspended in 450 μL staining buffer, consisting of 100 μL CellSearch nuclear stain, 100 μL CellSearch staining reagent, 100 μL CellSearch permeabilization reagent, 75 μL casein buffer and 75 μL PBS. After 20 min of staining at 37 °C 550 μL casein buffer was added and the sample was magnetically separated for 5 min in an iMAG magnet. After aspiration of the unbound fraction the sample was resuspended in 150 μL of CellSearch CellFix together with 175 μL PBS and placed into a CellSearch cartridge.

### 2.4 CTC and co‐enriched WBC enumeration

The CellSearch cartridges resulting from both CellSearch and FETCH processing were scanned using the CellTracks Analyzer II. The number of CTCs was determined using the celltracks software, while the number of co‐enriched WBCs as well as the number of CTC clusters was determined from the CellSearch archives using StarDist segmentation [10] in combination with a deep learning approach [16]. In the case of CTC clusters an operator manually reviewed the found clusters to prevent false positives.

### 2.5 Detection of clumping

In order to quantify the level of clumping present in the enriched samples, we evaluated for each sample 20 images from the CellTracks archive that did not contain cartridge borders. Using ImageJ (NIH, Bethesda, MD), the DAPI channel from each image was thresholded using the Triangle method after which the number of clumps was determined. Clumps were here defined as segmentations larger than 1500 pixels, representing 675 μm^2^.

### 2.6 Statistics

To be robust against the large variation in CTCs and co‐enriched WBCs, linear regression was performed after log‐transformation of the data. Levine's test was used to check normality after log‐transformation, showing adherence to a normal distribution for log‐transformed CTCs but not for WBCs nor large‐segmentation counts. CTC counts were therefore compared using paired t‐testing, while we used Wilcoxon signed ranks test to compare the number of co‐enriched WBC and large segmentations. For samples where the CTC count was 0, we have used 0.1 for display purposes as well as regression analysis and *t*‐testing and ignored these samples in the calculation of fold changes. All statistics were performed using Origin 2019B (OriginLab, Northampton, MA).

## 3 Results

### 3.1 Prevention of cell clumping

The main issue of immunomagnetic CTC enrichment from DLA samples is the large number of leukocytes that remain after immunomagnetic enrichment, hindering (automated) recognition of the CTCs. This issue is worsened by the commonly observed clumping and disintegration of leukocytes which further impede the enrichment and the identification of CTCs and CTC clusters. To investigate whether pre‐treatment of the DLA sample with DNase could improve the sample quality without CTC loss, we processed 20 2%‐DLA samples with and without DNase pre‐treatment.

**Fig. 2:**
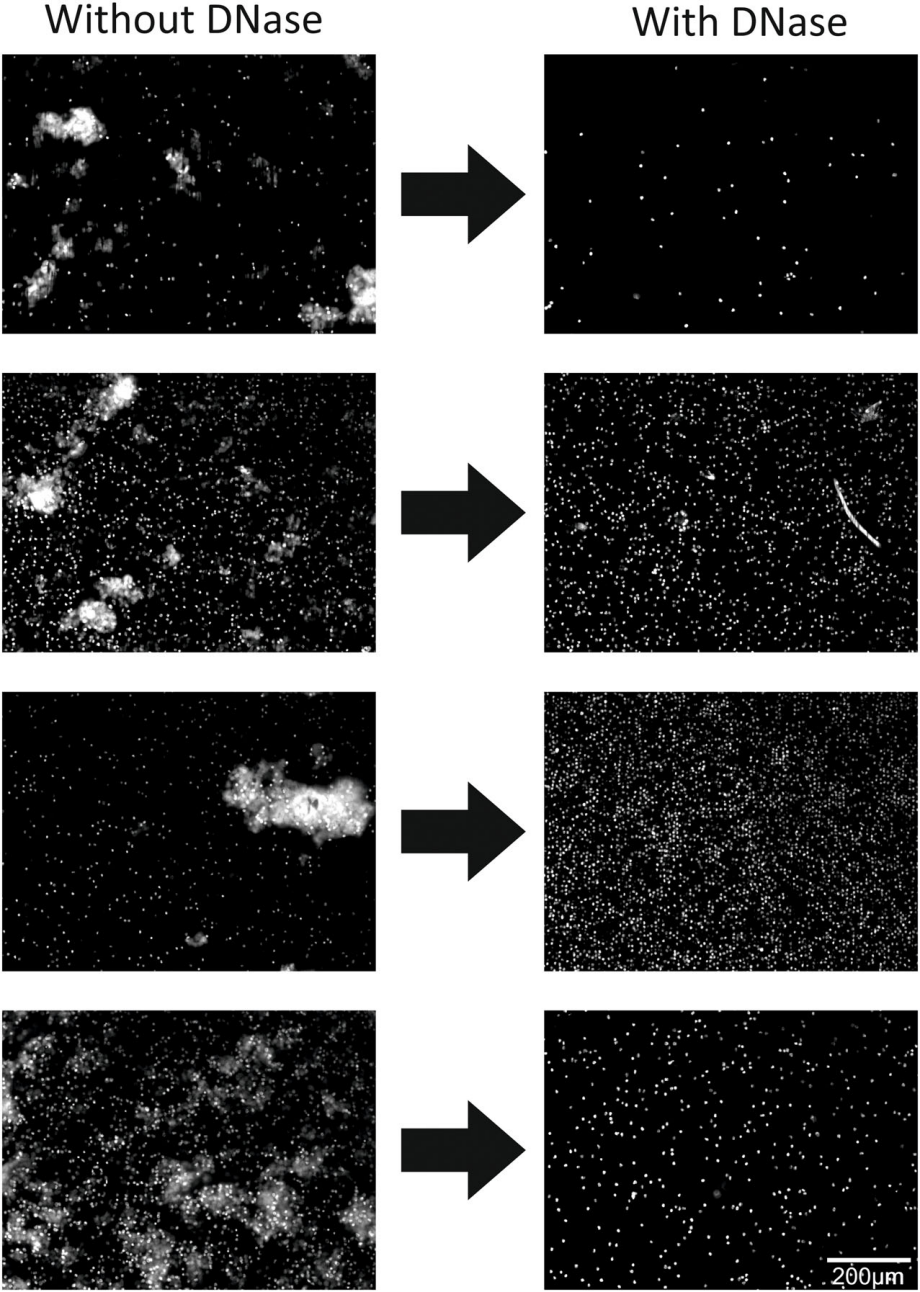
DNase treatment prevents cell clumping in CTC enrichment from DLA material. Representative DAPI fluorescent images of four sets of enriched samples obtained after the processing of DLA samples without and with DNase pre‐treatment, showing a clear decrease in cell clumping while the total number of nucleated events decreases for some and increases in other samples. The scalebar indicates 200 μm.

**Fig. 3:**
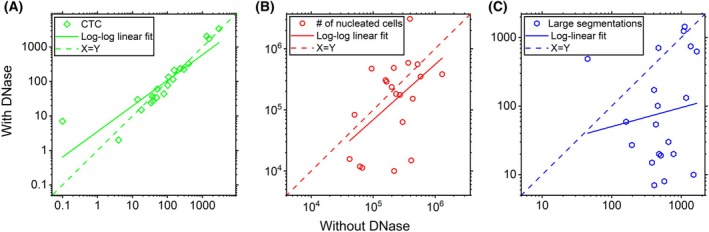
DNase pre‐treatment does not affect CTC recovery while greatly reducing cell clumping. (A) The number of CTCs, (B) the total number of nucleated cells, and (C) the number of large segmentations obtained after processing 20 DLA aliquots each consisting of 2% of the total samples using the FETCH with (*y*‐axis) or without (*x*‐axis) DNase pre‐treatment, showing no difference in the number of CTCs or the total number of nucleated cells, but a decrease in the number of excessively large segmentations due to the presence of cell clumps.

Figure 2 shows fluorescent images of the nuclear stain DAPI of four typical DLA samples after immunomagnetic enrichment of 2%‐DLA aliquots without (left) and with (right) DNase pre‐treatment. Whereas the untreated samples show leukocyte clumps and clouds of DAPI staining, these disappeared in the DNase pre‐treated samples. To evaluate the recovery of CTC, the leukocyte carry‐over and the number of clumps with and without DNase pre‐treatment were assessed in 20 immunomagnetically CTC‐enriched DLA aliquots from metastatic prostate cancer (mPC) patients. After enrichment the samples were fluorescently labeled and the number of CTCs, nucleated cells, and cell clumps were analyzed in the fluorescent images. Panel A of Fig. 3 compares the number of CTCs detected with and without DNase pre‐treatment. There is a single outlier where 0 CTCs were found in the no‐DNase sample and 7 CTCs in the DNase sample. All other data pairs are very close to the *x*–*y* line. A *t*‐test on log‐transformed data shows no statistically significant difference in the number of CTC found with or without DNase (*P* = 0.18). The linear regression is affected by the outlier and yielded log_10_(CTC_DNase_) = 0.55 + 0.74 log_10_(CTC_no‐DNase_) with an *R* ^2^ of 0.80. Panel B of Fig. 3 compares the number of nucleated cells after immunomagnetic enrichment with and without DNase. The correlation is weak (*R* ^2^ = 0.25), with a linear regression on log‐transformed data resulting in log_10_(WBC_DNase_) = −0.31 + 0.91 log_10_(WBC_No‐DNase_). Although we had hoped that the DNase pre‐treatment would also reduce the number of co‐enriched healthy cells, no statistically significant difference was found in this dataset (Wilcoxon Signed Ranks Test, *P* = 0.65). Panel C of Fig. 3 compares the number of large segmentations (> 675 μm^2^) in the DAPI channel with and without DNase. There is no correlation (*R* ^2^ = 0.02), and the occurrence of large segmentations is greatly reduced with the use of DNase pre‐treatment (Wilcoxon Signed Ranks Test, *P* = 0.001) as a result of the decrease in clumping. Although almost all clumps were prevented, a small number of small clumps could still be identified in the DNase pre‐treated samples, as for instance visible in the top right corner image on the second row of Fig. 2. Although the clumping that due to the presence of free‐DNA is fundamentally different from the naturally present CTC‐clusters, we evaluated the number of CTC‐clusters to assess if these are not broken up due to the DNase treatment. In total CTC‐clusters consisting of two or more CTCs were detected in samples from seven patients. Clusters were found in six samples without DNase pre‐treatment (median 3.5, range 1 to 9 clusters) as well as six samples (median 4, range 1 to 13 clusters) with DNase pre‐treatment. Although this dataset is too small for statistical significance, we did not find any indication that CTC‐clusters are broken up due to the DNase treatment. In light of this, we have used the FETCH system in combination with the DNase pre‐treatment in subsequent experiments.

### 3.2 FETCH vs CellSearch

**Fig. 4:**
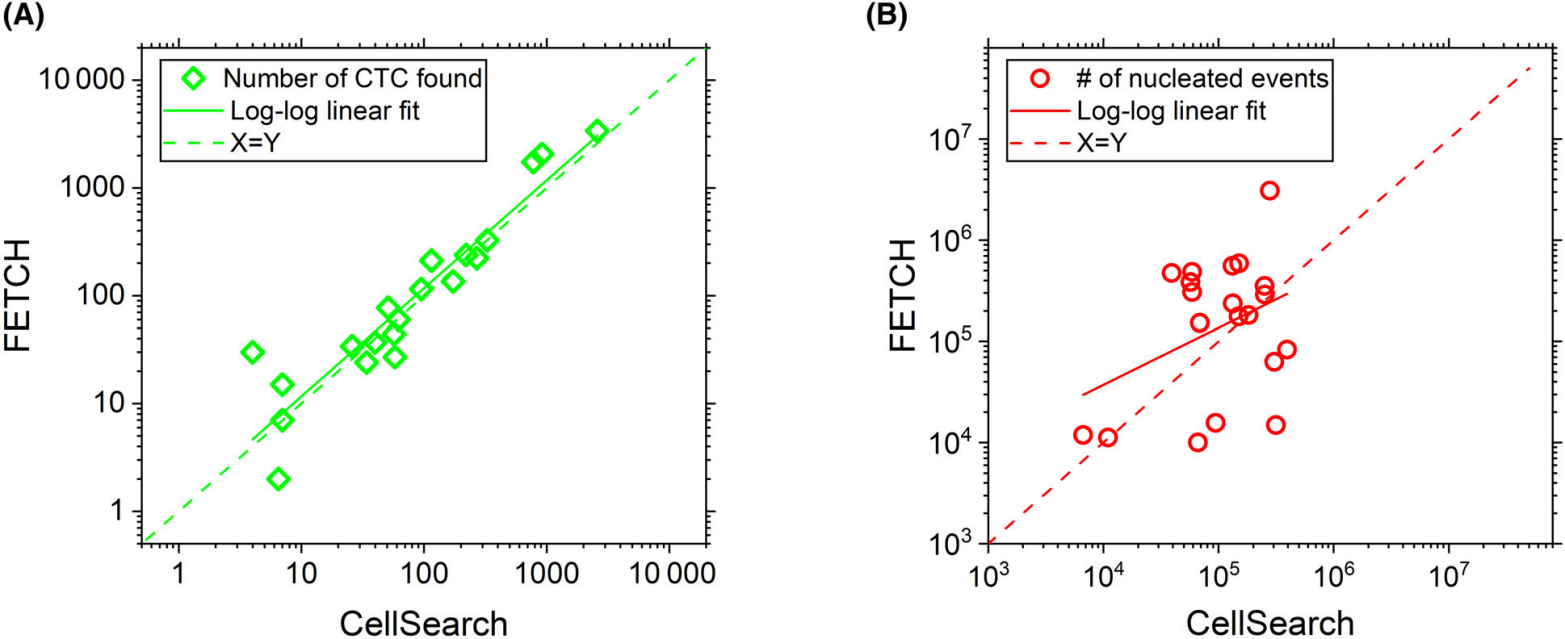
No CTC loss for FETCH enrichment compared to CellSearch. (A) The number of CTCs and (B) the number of nucleated events obtained from the processing of 20 DLA aliquots each consisting of 2% of the total sample (2%‐DLA) using CellSearch or the FETCH showing no loss in CTCs for the FETCH method.

To compare the CTC recovery and leukocyte co‐enrichment of FETCH‐based magnetic separation with that of CellSearch, we used 20 2%‐DLA aliquots of mPC patients. Fig. 4A shows the number of CTCs detected by FETCH versus CellSearch. No significant difference was found between the CTC counts of both methods (*t*‐test, *P* = 0.86). Linear regression analysis resulted in log_10_(CTC_FETCH_) = 0.06 + 1.00 log_10_(CTC_CS_) with an *R* ^2^ of 0.87, indicating no difference in CTC recovery between FETCH and CellSearch. Fig. 4B shows the number of co‐enriched WBCs during FETCH separation versus CellSearch. Here the correlation is weak (*R* ^2^ = 0.14), with a regression of log_10_(WBC_FETCH_) = 2.32 + 0.56 log_10_(WBC_CS_). Due to the increased magnetic force generated in the FETCH system, an increase in co‐enriched leukocytes is expected as a result of the higher sensitivity. However, the effect is not very strong in this dataset, with the median number of co‐enriched WBCs being 1.6‐fold higher when using FETCH processing, while the Wilcoxon Signed Rank test returns a *P* of 0.05. Also, the final sample purity for 2%‐DLA aliquots is similar for both methods. (Wilcoxon Signed Rank test, *P* = 0.62, see Fig. S1A).

### 3.3 FETCH for larger DLA aliquot processing

To test the potential of using FETCH for the processing of a 10‐fold larger DLA aliquot, we increased the fraction of the DLA sample per test from 2% to 20% through a doubling of the sample volume in combination with a five‐fold increase in the WBC concentration. This 20% of the DLA sample represents the product obtained from processing 1.1 liters of blood.

This increase in volume also resulted in the amount of capture reagents becoming equal to that normally used in the CellSearch processing; 150 μL of ferrofluid and 150 μL capture enhancement reagent.

**Fig. 5:**
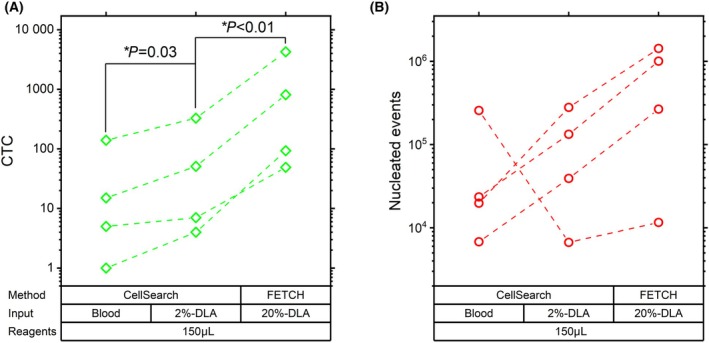
Increased obtainment of CTC by processing 10‐fold larger aliquots of DLA with FETCH. (A) The number of CTCs and (B) the total number of nucleated events obtained after the processing of 7.5 mL blood or DLA aliquots each consisting of 2% of the total sample (2%‐DLA) using CellSearch, or DLA aliquots consisting of 20% of the total sample (20%‐DLA) using the FETCH showing a significant increase in CTCs obtained by processing a larger aliquot of DLA (paired t‐test on log‐transformed data). All methods use the same volume of reagents. *N* = 4.

**Fig. 6:**
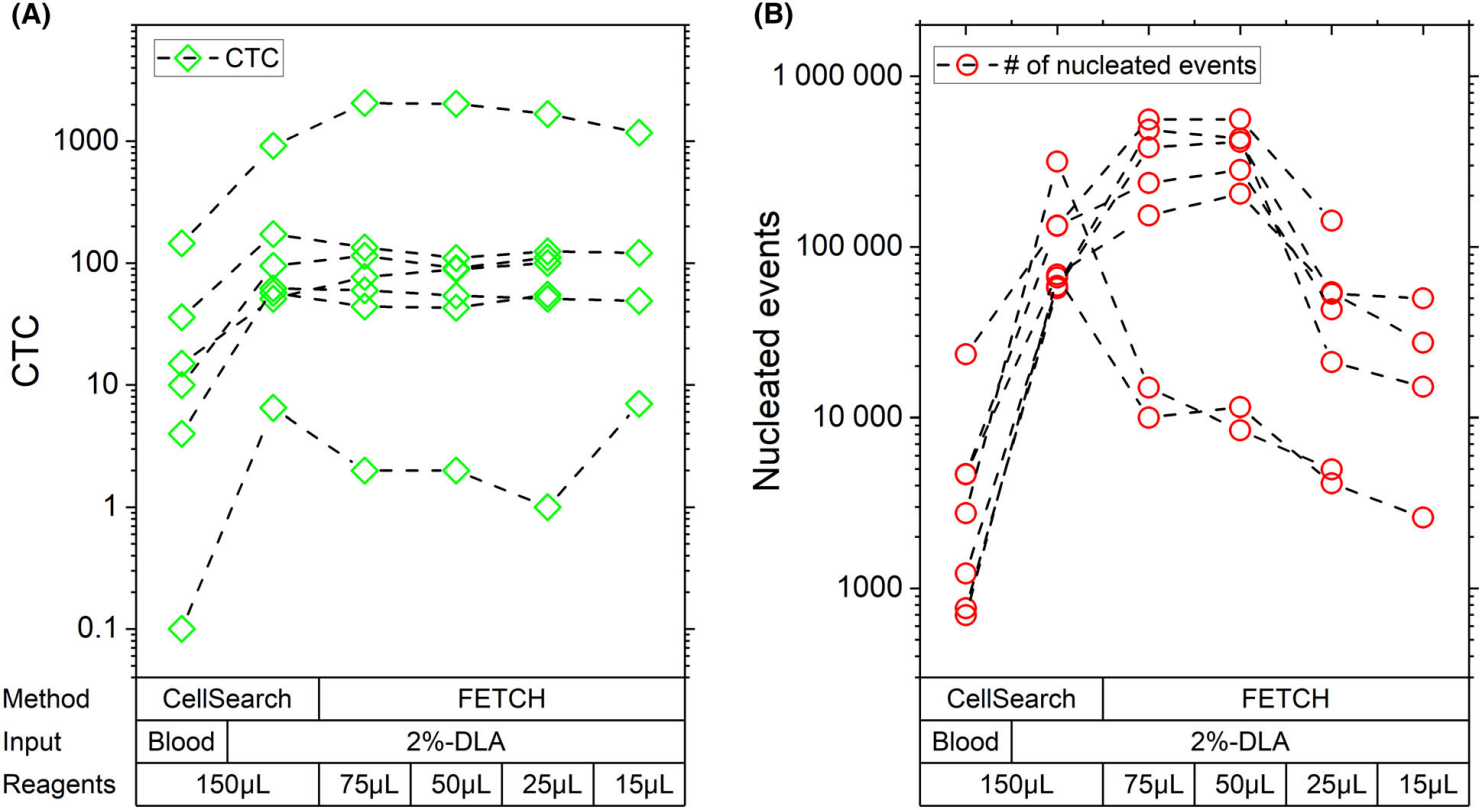
No loss in CTC but reduction in co‐enriched healthy cells when using FETCH with lower capture reagent volumes. (A) The number of CTCs and (B) the total number of nucleated events in the enriched sample obtained after processing 7.5 mL blood samples or DLA aliquots each consisting of 2% of the total sample (2%‐DLA) from mPC patients using CellSearch as well as the FETCH with 75, 50, and 25 μL (*N* = 7) as well as 15 μL (*N* = 4) of capture reagents. No loss in CTCs is seen as a result of the lower volume of capture reagents, while a decrease in the total number of co‐enriched nucleated events can be seen.

Using samples from four patients, we compared the CTC recovery, the total number of nucleated cells captured, and the resulting final sample purity when processing 7.5 mL blood samples and 2%‐DLA aliquots using CellSearch, with the processing of these 10‐fold larger 20%‐DLA aliquots using FETCH. Results in Fig. 5A show that in this limited sample set, after an initial median increase in CTCs of 2.8‐fold (mean 2.7, range 1.4 to 4.0‐fold) when moving from peripheral blood CellSearch to DLA processing with CellSearch, the processing of a 10‐fold larger DLA sample using FETCH resulted in an additional median 14.4‐fold (mean 14.8, range 7.0 to 23.3‐fold) increase in recovered CTCs (paired *t*‐test on log‐transformed data, *P* = 0.002). Together this results in a total median increase when compared to the processing of a 7.5 mL blood sample of 42.2‐fold (mean 46.8, range 9.8 to 93.0‐fold) while using the same reagent volume. The number of WBCs normally increases when moving from peripheral blood to DLA processing in CellSearch (see also Fig. 6), but as shown in Fig. 5B, in this subset one of the four samples showed a decrease in co‐enriched WBCs. For all four samples, an increase in co‐enriched WBCs was seen when moving from a 2%‐DLA aliquot processed with CellSearch to the processing of a 10‐times larger 20%‐DLA aliquot with FETCH. This increase of median 6.0‐fold (mean 6.3, range 1.7 to 7.6‐fold) was less than the increase in CTC, resulting in an increase in CTC purity. Nevertheless, despite the lack of clumps achieved through the use of DNase and the increase in CTC purity, with these 10‐fold larger samples, the WBC co‐enrichment still caused two to four CellSearch cartridges to be required per sample.

### 3.4 Reduced reagent concentration

We then postulated that as the increased WBC concentration for these 10‐fold larger samples did not reduce CTC capture, a reduction in the used reagent concentration might also be possible without any CTC loss, potentially improving the purity of the final samples due to a reduction of co‐enriched WBC. To test this, from seven patients we processed 7.5 mL blood samples as well as 2%‐DLA aliquots using CellSearch (standard capture reagent volume of 150 μL), and compared this to the processing of 2%‐DLA aliquots with FETCH using the standard 5 mL sample volume, but with capture reagent volumes of 75, 50, and 25 μL, while in four cases also an aliquot was processed using only 15 μL of capture reagent volume.

Results in Fig. 6A show that even when the reagent volume was decreased down to only 10% of the standard CellSearch reagent volume, no loss in CTC was found, with recovery remains equal to that of CellSearch (paired *t*‐test on log‐transformed data, *P* = 0.67). When reducing the reagent volume for FETCH to 25 μL, the number of co‐enriched WBC (Fig. 6B) sharply decreases, leading to a median 2.8‐fold (mean 12.6, range 0.9 to 63.4‐fold) decrease in co‐enriched WBC compared to CellSearch and a median 3.9‐fold (mean 6.8, range 2.4 to 23.0‐fold), decrease compared to FETCH with 75 μL of capture reagent. This trend seems to continue when further reducing the reagent volume to 15 μL, showing a median 3.2‐fold (mean 8.3, range 1.1 to 25.4‐fold) decrease in co‐enriched WBC compared to CellSearch and a median 6.6‐fold (mean 12.3, range 3.8 to 32.1‐fold) decrease compared to FETCH with 75 μL of capture reagent. As a result of this decrease in co‐enriched WBCs without a loss in CTCs, the CTC purity of the enriched samples increases, as seen in Fig. S1.

### 3.5 Increased sample concentration

**Fig. 7:**
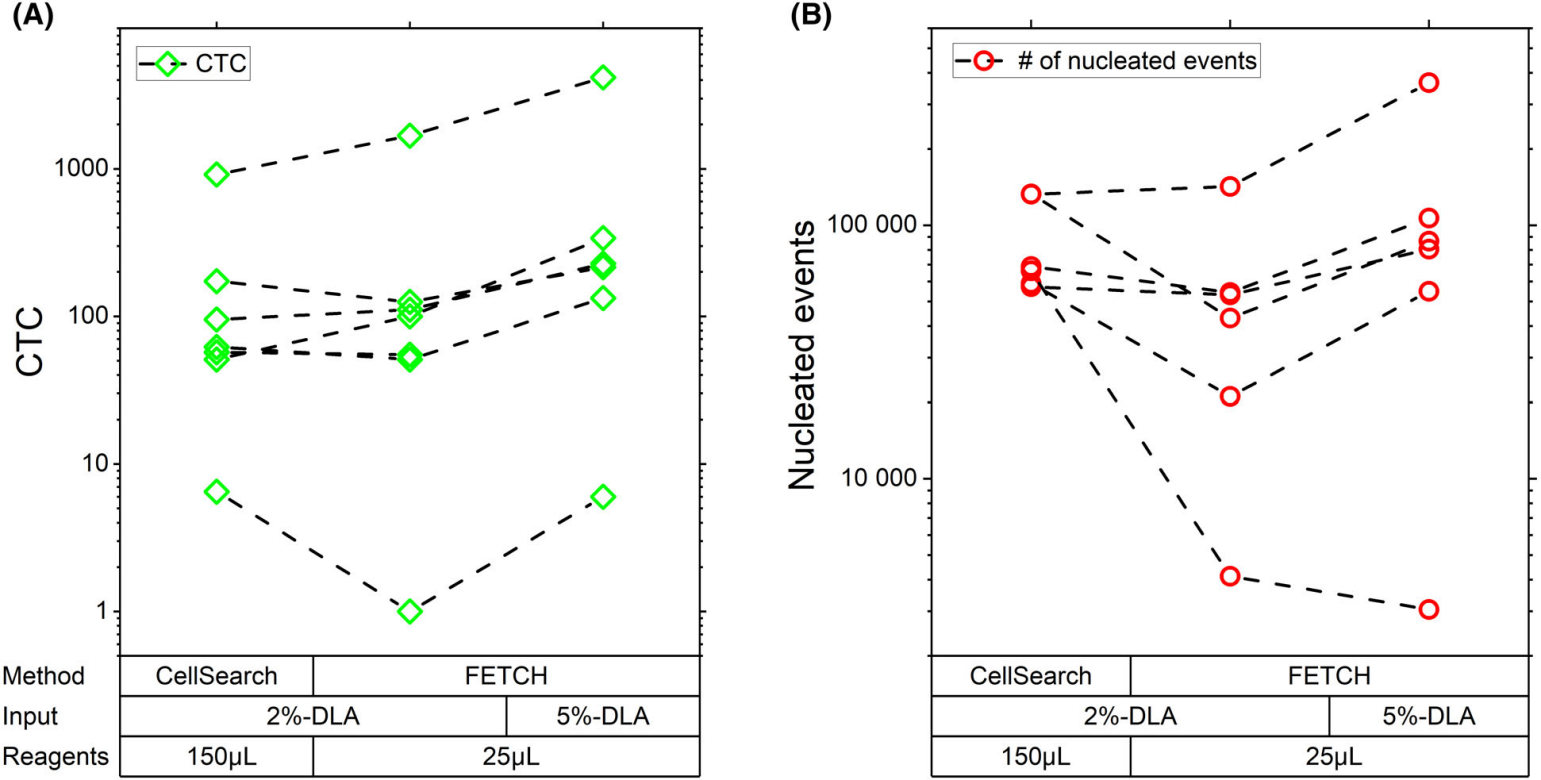
Combination of larger DLA aliquots with reagent volume reduction using FETCH shows an increased number of obtained CTC with a lower percentage of co‐enriched healthy cells. (A) The number of CTCs and (B) the total number of nucleated events in the enriched sample obtained after processing DLA aliquots each consisting of 2% (2%‐DLA) or 5% (5%‐DLA) of the total sample by CellSearch or the FETCH showing an equal increase in the number of CTCs obtained when processing a 2.5‐fold larger DLA aliquot with a reduced reagent concentration, whereas the number of co‐enriched nuclear events increase only 2‐fold. *N* = 7.

To examine the effect of increasing the WBC concentration at these reduced reagent volumes we processed from six patients 2%‐DLA aliquots as well as 5%‐DLA aliquots in a 5 mL volume. Results in Fig. 7A show that this 2.5‐fold increase in WBC input also resulted in a median 2.5‐fold (mean 3.0, range 1.7 to 6.0‐fold) increase in CTCs, resulting in a median 22.8‐fold (mean 22.7, range 6.0 to 33.3‐fold) increase in CTCs compared to the 7.5 mL blood sample. As a result, the obtained number of CTCs per sample input remained the same despite processing a 2.5‐fold larger input with the same reagent volume. The number of co‐enriched WBCs from a 2%‐DLA aliquot (Fig. 7B) showed a median decrease of 2.0‐fold (mean 4.2, range 0.9 to 16.0‐fold) when using FETCH with 25 μL of capture reagent compared to CellSearch, but increased again by a median 2.0‐fold (mean 1.9, range 0.7 to 2.6‐fold) when the sample input was increased to a 5%‐DLA aliquot, resulting in a slight increase in enriched sample purity (Fig. S1B).

## 4 Discussion

Since the introduction of DLA as a means of solving the low CTC frequency [6], the potential of this approach to obtain an increase of CTCs from patient samples has been demonstrated [7, 9, 17]. This has allowed the detection of CTCs in non‐metastatic cancer patients [6], the study of interpatient heterogeneity and tumor evolution [17] as well as for the generation of CTC derived cell lines [15, 18] and explants [19]. The high number of WBCs co‐enriched together with their clumping has caused most studies in which patient DLA material is processed for CTC enrichment use only small aliquots of DLA. Others have shown potential in processing larger DLA aliquots by combining RosetteSep depletion with positive CellSearch enrichment [9]. This approach however requires additional peripheral blood, is time and cost consuming, and the CTC yield compared to direct CellSearch is low. As a result of the decrease in reagent volume and increase in the WBC concentration that used in the FETCH system together with DNase pre‐processing presented here allows the processing of 20% of the DLA sample, representing over a liter of blood, without any CTC loss compared to 2%‐DLA processed with CellSearch. When processing 2%‐DLA aliquots down to 10% of the capture reagents can be used without CTC loss, resulting in a cost reduction, but also in a 3.3‐fold decrease in co‐enriched WBCs. While all results were obtained with CTCs in patient DLA products, and not using culture cells in a mock‐up DLA sample, they will need to be repeated on a larger number of samples as well as in different cancer types and stages.

Due to the clumping present in the samples without DNase pre‐treatment it is impossible to detect the occurrence of pre‐existing CTC‐WBC clusters, which have also been shown to be of importance [20]. It is expected that these CTC‐WBC clusters are not broken up due to the DNase pre‐treatment, as their interaction is not a result of free DNA, but of cell–cell junctions and cytokine‐receptor pairs [21]. This is supported by our finding that the number of CTC‐clusters does not appear to be affected by the DNase pre‐treatment. In this way, the prevention of clumping can improve the detection of true CTC‐WBC clusters. The processing of 10‐fold larger aliquots using FETCH was shown to be possible without CTC loss, potentially making the detection of CTCs in almost all metastatic cancer patients possible [5]. Although the prevention of cell‐clumps in these samples by DNase pre‐treatment makes both enumeration and single cell characterization more feasible, the sheer number of co‐enriched WBCs made the analysis of these samples challenging. As a result either a different analysis platform, such as the RareCyte [22] or HD‐SCA [23] platform, could be used, or a further reduction in co‐enriched WBCs is needed. Reducing also the reagent concentration when processing these highly concentrated samples could potentially provide this reduction, as indicated by our initial testing of processing a 2.5‐fold larger samples using only 25 μL of reagents. This combination of both sample increase and reagent concentration decrease resulted in both a larger total number of CTCs as well as an increased CTC purity, making downstream analysis less complicated. A further increase in WBC concentration and/or reduction of the reagent volume will need to be tested to determine the full possibilities of this approach.

## 5 Conclusion

White blood cell clumping during magnetic enrichment of CTC from DLA aliquots can be prevented by the use of DNase, without any loss of CTCs. Using the capture reagents of a single CellSearch test, 20% of a DLA sample can be processed by FETCH, resulting in a median 42‐fold increase in CTCs compared to a 7.5 mL blood sample. In the processing of 2%‐DLA aliquots, the co‐enrichment of WBCs can be decreased 3.2‐fold without CTC loss by the use of FETCH with only 10% of the regular volume of CellSearch capture reagents. By combining DNase pre‐treatment with an increase in DLA input and a decrease in reagent volume, the FETCH system allows for the (cost‐) effective enrichment of CTCs from DLA samples.

## Conflict of interest

MS and LT have filed a patent application based in part on this work and MS is a co‐founder of FETCH BV. ED and KI received funding from the NWO KWF PICTURES project, which is co‐funded by Menarini Silicon Biosystems and Terumo. LT and FC received funding from Menarini Silicon Biosystems, not in relation to this manuscript.

## Author contributions

Conceptualization, MS; Investigation, AM, FAWC, ED, KTI, JK & MS; Methodology, MS & LWMMT; Software, FAWC; Supervision, JWMM, RdW & LWMMT; Visualization, MS; Writing – original draft, MS & LWMMT; Writing – review and editing, all authors.

## Peer review

The peer review history for this article is available at https://www.webofscience.com/api/gateway/wos/peer‐review/10.1002/1878‐0261.13565.

## Supporting information


**Fig. S1.** CTC purity of enriched blood, 2% or 5% DLA samples processed CellSearch or FETCH.
**Table S1.** Differential blood and CTC count.

## Data Availability

All original data is available upon reasonable request.
