# Supplementary material for: Flow‐based immunomagnetic enrichment of circulating tumor cells from diagnostic leukapheresis product
Source: Mol Oncol. 2024 Jan 18;19(7):2133–43. doi: 10.1002/1878-0261.13565 (PMC12234375; doi:10.1002/1878-0261.13565)
Supplement: Supplementary file 1 — Fig. S1. CTC purity of enriched blood, 2% or 5% DLA samples processed CellSearch or FETCH. Table S1. Differential blood and CTC count. [file MOL2-19-2133-s001.pdf]

Flow-based immunomagnetic enrichment of circulating tumor cells from diagnostic leukapheresis product

M Stevens[1,2]*, A Mentink[1], FAW Coumans[1,3], E Dathathri1, KT Isebia[4], J Kraan[4], R de Wit[4], JWM Martens[4] and LWMM Terstappen[1,3,5]

[1] Department of Medical Cell BioPhysics, Faculty of Science and Technology, University of Twente, Enschede, The Netherlands
[2] FETCH BV, Deventer, The Netherlands
[4] Department of Medical Oncology, Erasmus MC Cancer Institute, Rotterdam, The Netherlands
[5] Department of General, Visceral and Pediatric Surgery, University Hospital Düsseldorf, 40225 Düsseldorf, Germany

Supporting information

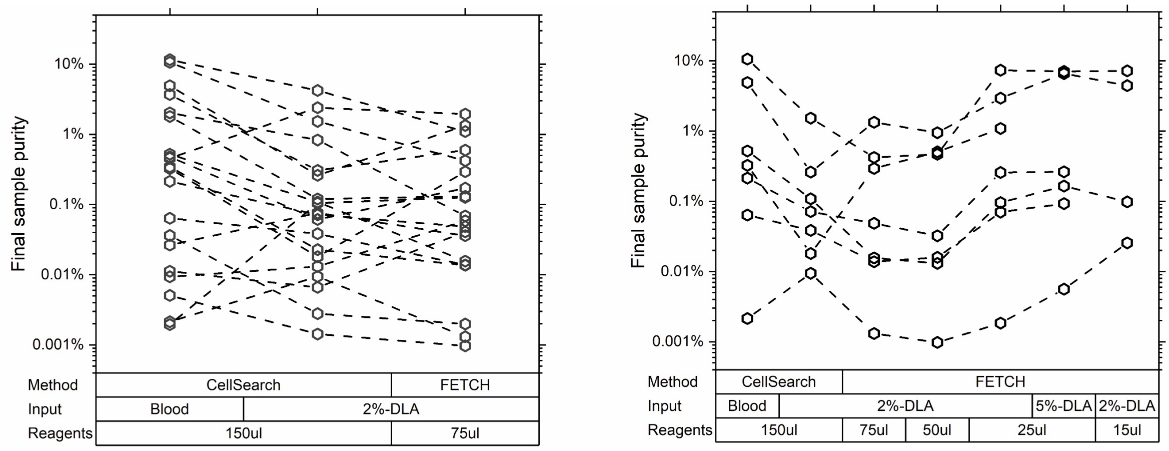

Figure S1; CTC purity of enriched blood, 2% or 5% DLA samples processed CellSearch or FETCH: circulating tumor cell (CTC) purity of the final samples obtained after enrichment of CTCs from A) 7.5 mL blood samples or diagnostic leukapheresis (DLA) aliquots each consisting of 2% of the total sample samples using CellSearch or the flow enrichment target capture Halbach-array (FETCH) (N=20) and B) 7.5 mL blood samples (N=7) or 2%-DLA samples using CellSearch or FETCH with 75, 50, 25 (N=7) or 15 µl (N=4) of reagents as well as 5%-DLA using 25 µl of reagents (N=7).

Sample#	Erythrocytes (10^6/ml)	Lymphocytes (10^6/ml)	Monocytes (10^6/ml)	Neutrophils (10^6/ml)	Eosinophils (10^6/ml)	Basophils (10^6/ml)	Platelets (10^6/ml)	Product (mL)	CTC/ ml
1	600	109,1	47,7	3,7	0	0,2	1839	81	217
2	360	69,92	33,28	19,67	0,02	1,03	2844	99	21
3	320	46,25	20,44	15,88	0,07	0,52	1682	102	11
4	370	33,18	15,98	8,78	0,02	0,19	1601	94	17
5	380	48,06	22,86	31,72	0,06	0,48	2594	113	169
6	490	42,49	22,42	73,86	0,7	0,84	1788	91	5
7	410	43,04	31,93	30,56	0,13	0,78	1674	77	415
8	390	62,39	25,05	0,14	0,01	0,03	1420	76	1136
9	550	39,53	27,99	8,5	0,02	0,52	1959	97	15
10	360	58,11	31,91	7,81	0,13	0,53	1562	90	3
11	430	53,69	18,57	3,67	0,02	0,34	2035	98	84
12	420	67,11	36,36	2,67	0,01	0,42	3890	79	30
13	460	53,39	24,4	2,12	0,05	0,22	1217	78	46
14	420	41,28	35,62	64,32	0,47	0,00	1773	116	3
15	270	40,18	20,21	0,83	0,01	0,15	1696,4	93	29
16	300	57,1	31,5	58,3	0,4	0,9	2111	111	38
17	320	48,77	17,49	9,82	0,01	0,24	1776,3	91	350
18	430	43,27	21,42	1,96	0,01	0,15	2617,3	97	2
19	330	57,14	17,69	11,93	0,07	0,67	3153	89	27
20	490	74,23	45,31	25,48	0,15	1,04	2181,5	76	126
Mean	405	54,41	27,41	19,09	0,12	0,46	2070,68	92	137
Median	400	51,08	24,73	9,3	0,035	0,45	1813,65	92	30
Min	270	33,18	15,98	0,14	0	0	1216,7	76	2
Max	600	109,1	47,7	73,86	0,7	1,04	3890	116	1136

Supplementary table S1; Differential blood and CTC counts: Differential blood count of the obtained diagnostic leukapheresis (DLA) products, total volume of DLA obtained as well as the number of circulating tumor cells (CTCs) per mL of DLA based on the CTC detection by CellSearch.
